# Thoracic wall blocks in cardiac and thoracic procedures: expanding frontiers for perioperative regional analgesia

**DOI:** 10.1016/j.bjane.2025.844670

**Published:** 2025-08-13

**Authors:** Marcello Fonseca Salgado-Filho, Luiz Guilherme Villares da Costa, Eric Benedet Lineburger, Bruno Francisco de Freitas Tonelotto

**Affiliations:** a Hospital Israelita Albert Einstein, Departamento de Anestesiologia, São Paulo, SP, Brazil; b Hospital São José, Departamento de Anestesia e Tratamento da Dor, Criciúma, SC, Brazil; c Hospital Sírio-Libanês, São Paulo, SP, Brazil

Regional anesthesia techniques have gained increasing relevance in cardiac and thoracic surgeries, driven by the pursuit of more effective analgesic strategies, reduced opioid use, and improved postoperative recovery. Traditionally, pain management in surgeries involving thoracotomy or sternotomy relied on intravenous opioids and, in some cases, neuraxial techniques such as thoracic epidural blocks. Although cardiothoracic procedures have advanced significantly toward minimally invasive techniques, such as video-assisted and robotic surgeries and despite the smaller incisions compared to sternotomy and thoracotomy, these approaches are still associated with significant postoperative acute pain and a portion of patients may develop persistent chronic pain that lasts for months or even years after the procedure.^1^, ^2^, ^3^

The Enhanced Recovery After Cardiac Surgery (ERACS) protocols recommend the use of multimodal strategies for pain management, highlighting the role of myofascial blocks in reducing opioid requirements in the perioperative period. This has led to the growing use of thoracic wall fascial plane blocks, such as the erector spinae plane (ESP) block, transversus thoracic muscle plane (TTMP) block, and pectointercostal fascial (PIF) block.^1^, ^2^ While observational studies and case series suggest their effectiveness, significant gaps remain regarding the superiority of specific techniques, their safety profiles in specific populations, long-term clinical outcomes, and cost-effectiveness.^1^, ^2^, ^3^

In the setting of adult cardiac surgery, regional techniques are increasingly valued as part of multimodal strategies for enhanced recovery. Recent studies demonstrate that fascial plane blocks can significantly reduce opioid consumption, shorten the duration of mechanical ventilation, and facilitate early discharge from the intensive care unit (ICU), even in patients undergoing median sternotomy.^1^, ^2^ Kelava et al. highlighted the applicability of ESP, PIF, and TTMP blocks,^1^ while Rubio et al. advocated for their routine adoption to support fast-track cardiac anesthesia programs.^2^ Conversely, Jia et al. raised concerns about methodological limitations in the current literature, implementation costs, and logistical barriers, calling for caution before generalizing these techniques to all adult cardiac procedures.^3^

Within this debate, Damião et al., published in the Brazilian Journal of Anesthesiology (BJAN), reinforce the benefit of ultrasound-guided ESP block by demonstrating reduced pain scores and morphine consumption after sternotomy.^4^ In congenital cardiac surgeries, thoracic wall blocks have also gained attention. A recent meta-analysis published in this BJAN issue evaluated the impact of ESP block in children undergoing cardiac surgery, demonstrating consistent reductions in postoperative pain and opioid requirements within the first 24 hours.^4^ Notably, a Bayesian network meta-analysis by Ren et al. compared multiple pediatric regional techniques and found ESP to be the most effective overall, despite moderate-quality evidence.^5^ Interestingly, another Bayesian Network meta-analysis identified TTMP as a consistently top-performing technique across outcomes.^6^ This scenario is particularly promising in pediatrics due to the favorable safety profile of fascial plane blocks and the known vulnerability of pediatric patients to the adverse effects of opioids. Damião et al. contributed original clinical data on bilateral ESP blocks in pediatric cardiac surgeries, reinforcing their safety and analgesic efficacy.^4^ Complementing these findings, Ali Gado et al. conducted a randomized controlled trial specifically examining bilateral ESP blocks in children undergoing cardiac surgery via median sternotomy.^7^ Their results confirmed the analgesic efficacy and safety of the technique, highlighting reduced pain scores at rest and during coughing, and supporting the use of ESP as a key component of multimodal analgesia protocols in this population.

In thoracic surgeries, several studies have demonstrated effective analgesia with blocks such as the ESP block, thoracic paravertebral block, and serratus anterior block.^8^, ^9^ The PROSPECT group (Procedure-Specific Postoperative Pain Management) recommends, with level A evidence, thoracic epidural anesthesia or paravertebral block as the techniques of choice for postoperative analgesia.^9^, ^10^ However, despite its popularity, the ESP block exhibits erratic spread of local anesthetic, as shown in cadaveric studies and clinical practice, leading to block failures.^8^

In 2023, Tulgar et al. described the superior posterior serratus intercostal plane block (SPSIPB), demonstrating homogeneous spread of local anesthetic toward upper thoracic and cervical fibers in cadaver studies.^11^ This technique is applicable to thoracic, breast, and shoulder surgeries. In this BJAN issue, Doğan et al. conducted a prospective randomized clinical trial comparing SPSIPB with thoracic paravertebral block in patients undergoing video-assisted thoracic surgery (VATS).^11^ Results showed that SPSIPB was non-inferior to the paravertebral block, offering similar analgesic efficacy, despite the paravertebral block showing lower pain scores only in the first postoperative hour.^11^

In both thoracic and cardiac surgeries, Enhanced Recovery After Surgery (ERAS) protocols have driven transformative changes in perioperative care by emphasizing multimodal analgesia, early extubation, rapid mobilization, and reduced hospital length of stay.^1^, ^2^ The integration of fascial plane blocks into ERAS and ERACS pathways is a growing trend, offering promising improvements in patient satisfaction, respiratory outcomes, and health care costs. Nevertheless, the success of these protocols depends on local infrastructure, the availability of trained personnel, team engagement, and appropriate case selection.^1^, ^2^, ^3^ Makkad et al. emphasized that regional techniques should be integrated contextually, tailored to institutional capabilities, and continuously evaluated for clinical impact.^8^

In summary, thoracic wall fascial plane blocks are emerging as valuable tools for postoperative analgesia in cardiac and thoracic surgeries. Their widespread implementation will depend on higher-quality evidence, especially from randomized controlled trials assessing functional outcomes. The articles presented in this BJAN issue contribute significantly to the field by providing data supporting the efficacy and feasibility of myofascial blocks across age groups and surgical settings. Future research priorities include standardizing technique, elucidating the mechanism of action, evaluating long-term outcomes, and incorporating these strategies into value-based perioperative care. We are in a good time to consolidate these techniques with scientific rigor.

## Conflicts of interest

The authors declare no conflicts of interest.
